# Health Status of Adults with Hearing Loss in the United States

**DOI:** 10.3390/audiolres11010011

**Published:** 2021-03-10

**Authors:** Jennifer Glassman, Timothy Jordan, Jiunn-Jye Sheu, Lori Pakulski, Amy Thompson

**Affiliations:** 1 School of Intervention and Wellness, University of Toledo, Toledo, OH 43606, USA; lori.pakulski@utoledo.edu; 2 School of Population Health, University of Toledo, Toledo, OH 43606, USA; Timothy.Jordan2@utoledo.edu (T.J.); jiunnjye.sheu@utoledo.edu (J.-J.S.); amy.thompson4@utoledo.edu (A.T.)

**Keywords:** hearing loss, health behaviors, health status, disease state

## Abstract

Purpose: The purpose of this study was to identify the current health status of adults in the United States with self-reported hearing loss and compare it with US adults with a self-reported excellent or good hearing in three areas: (1) chronic disease states and general health status, (2) medical screening behaviors, and (3) lifestyle behaviors. Methods: A secondary data analysis was conducted using the 2014 data set from the National Health Interview Survey (NHIS), specifically the Sample Adult Public Use File (samadult). For this questionnaire set, one adult per family was randomly selected. This individual self-reported their response to the questionnaire items. Binary regressions were used to analyze the odds ratio to find differences for selected disease states, screenings, and lifestyle behaviors. Respondents were grouped into one of four categories: excellent/good hearing, a little trouble hearing, moderate/a lot of trouble hearing, and deaf. Results: The excellent/good hearing group was used as the comparison group for the other three levels of hearing. There are many differences in likelihood to self-report disease states; the greatest increased likelihoods include tinnitus and heart disease, with tinnitus being 8.6 times more likely for those who identified as having moderate/a lot of hearing loss. Those with any level of hearing loss were 3 to 5 times more likely to self-report heart disease. Regarding lifestyle factors, individuals with any level of hearing loss were less likely to consume alcohol and 2.5 to 9 times more likely to be unable to engage in moderate or vigorous activity on a weekly basis, respectively. Conclusions: There is a difference in the health status of individuals with hearing loss across all three areas examined (chronic disease states and general health status, medical screening behaviors, and lifestyle behaviors), and those differences vary based on level of hearing loss, the most notable being the self-reported inability to engage in moderate and vigorous physical activity. Disproportionate rates of tinnitus and heart disease were evident in all levels of hearing loss but most notable in those identifying as having moderate/a lot of trouble hearing. Further interdisciplinary research is necessary to improve the health of individuals with all levels of hearing loss, increase awareness of the hearing/health connection, and decrease hearing loss in general.

## 1. Introduction

Approximately 38 million adults in the United States (approximately 15% of adults) suffer from some degree of hearing loss [1]. The number of Americans with hearing loss continues to increase [2]. This number will continue to grow as the population of the United States ages and as hearing loss in American youth continues to increase. From 1988 to 2006, there was a 4.6% increase in hearing loss among US adolescents (14.9% to 19.5%) [3]. Experts estimate that more than 73 million American adults will have hearing loss by the year 2060 [4].

Although the number of Americans with hearing loss is increasing, little is known about the health status of adults with hearing loss. Even less is known about the association between hearing loss and health behaviors. In 2008, Schoenborn and Heyman used data from the National Health Interview Survey (NHIS (2000–2006)) to identify some aspects of health status for adults with hearing loss. They reported that adults who were deaf or had a lot of trouble hearing were almost three times more likely than those with normal hearing to rate their health as fair or poor. Furthermore, adults with deafness or significant hearing loss had higher rates of hypertension and diabetes and were more likely than those with normal hearing to engage in certain health risk behaviors (e.g., smoking, high-risk drinking, reduced physical activity, obesity, and reduced sleep) [5].

Other researchers have also reported associations between hearing loss and specific disease states. For example, diabetes is positively associated with hearing loss [6,7]. In one study, having diabetes for more than 10 years was associated with an increased level of hearing loss [6]. Cardiovascular risk factors such as high blood pressure and obesity have also been positively associated with hearing loss [8,9,10]. 

Some researchers have also reported a positive association between chronic kidney disease and hearing loss, particularly sudden sensorineural hearing loss [11,12]. Lin et al. (2013) also found that the co-occurrence of kidney disease and diabetes increased the risk of sensorineural hearing loss. Hearing loss has also been shown to have a negative impact on an individual’s social and cognitive status [5,13,14,15]. In contrast to the research above, Shargorodsky et al. (2010) found no association between hypertension and hearing loss [3]. 

Unfortunately, there is a paucity of research regarding the association between hearing loss and specific health behaviors. The few studies that do exist reveal interesting results. For example, smoking seems to have a negative effect on hearing [3,8,16,17,18]. In contrast, alcohol consumption, with the exception of life-long abstainers, was found to be protective of hearing [16,19,20].

One reason for the difference in health status between those with hearing loss and those with normal hearing may be differences in health behaviors between these two groups. If hearing loss is positively associated with negative health status and health risk behaviors, it is vital that public health experts, audiologists, and healthcare providers find ways to intervene and prevent deleterious health outcomes among those with hearing impairments. Therefore, the purpose of this study is to more fully investigate the association between hearing loss, health status, and health behaviors. 

## 2. Materials and Methods

### 2.1. Study Design and Participants

This study was an observational study using secondary data analysis methods to analyze the responses from the 2014 National Health Interview Survey (NHIS). The NHIS is an annual survey conducted by the US Census Bureau using a face-to-face interview format in participants’ homes. Hence, participants in the current study were non-institutionalized adults in the United States aged 18 to 85 who participated in the 2014 NHIS. The survey required approximately one hour to complete [19]. 

### 2.2. Sampling

A total of 36,697 interviews were completed from the 44,552 participating households in the 2014 NHIS. The survey results were labeled “Sample Adult” by the Census Bureau. The response rate for the “Sample Adult” interviews was 80.5%. Sample weighting was based on the 2010 census population estimates. The random sample used by NHIS came from all 50 states and the District of Columbia and was based on the four census regions. Within census regions, participants were selected from both metropolitan and non-metropolitan areas. The sampling design featured a complex, multistage sample method that included stratification, clustering, and oversampling of selected subgroups [19].

### 2.3. Instrumentation 

The interview guide/survey for adults was called the “Sample Adult Questionnaire”. The accompanying data file (samadult) released by the NHIS (identifying information removed) had 769 possible questions. The adult survey included 8 sections. The section titles and the number of questions in each section were as follows: (1) adult identification (AID, *n*= 4), (2) adult socio-demographics (ASD, *n* = 16), (3) adult conditions (ACN, *n* = 282), (4) adult health status and limitations of activity (AHS, *n* = 235), (5) adult health behaviors (AHB, *n* = 49), (6) adult healthcare access and utilization (AAU, *n* = 145), (7) adult selected items (ASI, *n* = 31), and (8) adult internet and email usage (AWB, *n*= 7). 

To assess hearing status, participants were given the following directions and were asked these questions: “These next questions are about your hearing without the use of hearing aids or other listening devices. Is your hearing excellent, good, a little trouble hearing, moderate trouble hearing, a lot of trouble hearing, or are you deaf?” The item that assessed overall health status was: “Compared with 12 months ago, would you say your health is better, worse, or about the same?” 

Self-reported health indicators, such as disease states or health conditions, were asked with the question stem, “Have you ever been told by a doctor or other health professional that you had…?” Slight variations occurred with the question regarding diabetes, “Other than pregnancy, have you ever been told by a doctor or other health care professional that you had diabetes or sugar diabetes?”, “Have you ever been told by a doctor or other health care professional that you had cancer or a malignancy of any kind?”

### 2.4. Data Analysis 

The investigators of the current study used the 2014 NHIS data file for adults. To facilitate comparisons of respondents with different hearing levels, the investigators grouped the adult respondents into four subgroups based on self-reported hearing acuity: (1) excellent/good hearing (normal), (2) a little trouble hearing, (3) moderate trouble/a lot of trouble hearing, and (4) deaf. 

We analyzed these four hearing groups in relation to their health status indicators and health behaviors. We examined three categories of health indicators: (1) self-reported health status (disease states), (2) self-reported medical behaviors (preventive care), and (3) self-reported health behaviors (lifestyle behaviors). The disease states that we examined included diabetes, hypertension, obesity, stroke, chronic obstructive pulmonary disease (COPD), coronary heart disease, hypercholesterolemia, and cancer. The medical/preventive care behaviors that we examined included various screening tests and vaccinations. The health behaviors that we examined included tobacco use, alcohol use, and physical activity. 

Descriptive statistics such as mean and standard deviations were used to describe the respondents and their responses on various survey items. Binary regression was used to assess the association between the hearing variables and the health-related outcomes. All analyses were completed using Statistical Product and Service Solutions (SPSS) 23 [20]. The a priori alpha level was set at <0.05. 

## 3. Results

### 3.1. Participants

Participants in the 2014 NHIS were sampled from the four census regions: South (35%), West (27%), Midwest (21%), and the Northwest (16%). Participants were White (78%), over age 40 (66%), female (55%), with excellent hearing (48%), and married and living with a spouse (42%). About 1 in 5 adults (19%) reported some level of hearing loss (Table 1). Regarding health, the majority of these respondents considered their health status to be about the same as it was last year (74%), and nearly one out of every 10 participants (9%) reported worse health than the previous year. Nearly two thirds of respondents (65%) were overweight or obese. Approximately a third of the respondents reported having hypertension and high cholesterol; other chronic diseases were less prevalent (Table 2). 

### 3.2. Self-Reported Health Status by Hearing Level 

Adults with all levels of hearing loss were less likely to rate their health as “about the same as last year” than individuals with excellent/good hearing. Participants with moderate hearing loss/a lot of trouble hearing were almost 3× more likely (OR = 2.93) to rate their health as “worse” than last year compared to those with excellent or good hearing. Individuals who were deaf were slightly more likely (OR = 1.33) to rate their health as “better” than last year compared to those with excellent/good hearing (Table 3).

#### Disease States and Hearing Level

Degree of hearing loss was associated with the following disease states: tinnitus, heart disease, cancer, COPD, diabetes, ulcers, hypertension, stroke, and high cholesterol. Rates of ulcers, hypertension, diabetes, and cancer increased as degree of hearing loss increased ranging from 2 times (OR = 2.21 for ulcers) to 3.5 times (OR = 3.72 for cancer) more likely to occur when compared to excellent hearing comparison group. Adults reporting “moderate/a lot of trouble hearing” were 5 times (OR = 5.05) more likely to experience heart disease and had the highest reported levels of tinnitus. This group also had the greatest increased risk (3–5×) of COPD (OR = 4.83), stroke (OR= 4.44), and hypertension (OR = 3.43). Individuals identifying as deaf were in the lowest risk group for COPD (OR = 2.59), stroke (OR = 2.05), and hypertension (OR = 3.38), but still at least twice as likely as the comparison group (excellent/good hearing) to have the aforementioned chronic diseases (Table 4).

The likelihood of having tinnitus was much greater for those with any level of hearing loss but did not consistently increase as hearing difficulty increased. Adults who reported hearing loss of any level were between nearly 4 to 9 times (OR= 3.9 to OR = 8.66) more likely to report experiencing tinnitus than their peers with excellent/good hearing (Table 3). Individuals reporting moderate/a lot of trouble hearing had the highest likelihood of tinnitus.

### 3.3. Medical Screening and Preventive Behaviors by Hearing Loss

Participants with any level of hearing loss were less likely to have seen a dentist in the past 12 months (OR = 0.76–0.80); however, they were up to twice as likely to have completed other preventative health screenings the last year when compared with participants with excellent/good hearing (Table 4) depending on the screening. In addition, with the exception of deaf males who were more likely to receive the HPV vaccine (OR = 1.47), all hearing loss levels were less likely (OR = 0.35 to 0.66) to report ever receiving an HPV vaccination than participants reporting excellent/good hearing (Table 4).

HIV testing was low across all participants, with slightly more than 1/3 reporting they had been tested for HIV (Table 5). Men who were deaf (OR = 0.50) and women reporting moderate/a lot of trouble hearing (OR = 0.49) were half as likely to have been tested for HIV as their excellent/good hearing counterparts (Table 5).

### 3.4. Health Behaviors (Lifestyle Behaviors) and Hearing Loss

Regarding lifestyle behaviors of all the participants, many consumed alcohol (63%), had never smoked cigarettes (60%), were sleeping 7–8 h on average each night (58%), and were engaging in moderate physical activity on a weekly basis (58%) (Table 6).

Participants with hearing loss varied in their lifestyle (health) behaviors. Overall, those with any level of hearing loss had similar hours of sleep, were about 50% less likely to report never being a smoker (OR = 0.48–0.64), and were much less likely to consume alcohol (12+ drinks a year) (OR = 0.05–0.06) than those reporting excellent/good hearing (Table 7).

Respondents with a little trouble hearing had similar alcohol consumption rates as those with excellent/good hearing but were 2.5 times more likely than those with excellent/good hearing to report difficulty with engaging in moderate (OR = 2.71) and vigorous physical activity (OR = 2.54) (Table 7).

Individuals reporting moderate/a lot of trouble hearing were twice as likely (OR = 2.23) to get more than 8 h of sleep per night. They were also more than twice as likely than as adults with excellent/good hearing to be former smokers (OR = 2.44). With exercise, however, they were more than 4 times more likely than those with normal hearing to report being unable to engage in moderate exercise (OR = 4.19) and vigorous exercise (OR = 4.15) (Table 7).

Participants identifying as deaf also reported being three times more likely (OR = 3.01) to get more than 8 h sleep than adults who identified as having excellent/good hearing. They were also almost half as likely to consume alcohol less than 1 day a week (OR = 0.55). Finally, most dramatically different were the exercise habits of people who were deaf. This group was more than 9 times more likely than those with normal hearing to report not being able to engage in moderate physical activity (OR = 9.45) and 7 times less likely to not engage vigorous activity (OR = 7.03) (Table 7). 

## 4. Discussion

The purpose of the current study was to investigate the association between hearing loss, health status, and health behaviors. Overall, hearing loss was associated with greater risk of self-reported disease and poor health status. Those who were deaf reported their health as “worse” than last year three times more often those with excellent/good hearing. For most of the disease states assessed, individuals with any level of hearing loss had a greater likelihood of disease when compared to adults with excellent/good hearing. For example, individuals with moderate/a lot of difficulty hearing had a greater likelihood of being diagnosed with COPD, stroke, high cholesterol, and tinnitus compared to adults in other hearing acuity categories. Furthermore, people with any level of hearing loss reported suffering from tinnitus and heart disease at disproportionately high rates compared to those with excellent/good hearing status.

The positive association between hearing loss and disease was a noteworthy finding. The heaviest disease burden seemed to fall upon individuals with moderate/a lot of hearing loss—even more so than those who were deaf. Adults with moderate/a lot of hearing loss were most likely to report having hypertension, COPD, stroke, high cholesterol, ulcers, hepatitis, long-term liver conditions, and tinnitus than individuals at any other level of hearing loss. It should be noted that tinnitus itself is associated with noise-induced hearing loss, hormone changes, and heart disease [21]. In contrast, those who reported themselves as deaf had the greatest likelihood of having diabetes and cancer (any kind) when compared to all levels of hearing.

Potential reasons for such differences in self-reported disease by hearing level may be the result of increased communication barriers faced by those with hearing loss when they seek medical care and obtain follow-up care with specialty physicians. Adults with hearing loss may be less motivated to seek medical care and/or seek it less frequently because of their tendency to withdraw socially, avoid engagement with others, and to disengage from conversations/situations they are already engaged in [22]. Patients with hearing loss can also experience communication problems during physical exams, during procedures, and with office staff, and have concerns about medication and safety [23]. Iezzoni and colleagues (2004) also reported that physicians may view patients with hearing loss are disengaged and/or unintelligent [24]. Surely, such perceptions are noticed by patients with hearing loss. Barriers like these can certainly make adults with hearing loss reluctant to seek medical care or to seek it less frequently than adults with normal hearing, potentially leading to late diagnosis and/or treatment.

Such findings regarding physicians’ negative perceptions of adults with hearing loss point to the need for improved physician training during medical school and residency [24,25]. Specialized training will likely need to involve more exposure to and experience with deaf patients and patients with moderate to severe hearing loss. One possibility would be for medical schools and residency programs to work with local audiology practices to recruit patients who have significant hearing loss. Such patients could then be trained as standardized patients to help medical students and residents to learn important adaptations that are needed to effectively communicate with hard-of-hearing or deaf patients.

Difficulties with comprehension and memory may also be a contributing factor to the higher self-reported disease rates reported by adults with hearing loss. Hearing acuity and memory have been shown to have an inverse association [26]. Tun and colleagues (2009) reported that adults with hearing loss have reduced comprehension of medical visits and less accurate recall of information or directions provided to them by healthcare providers [27]. Hence, adhering to medication regimens, therapeutic regimens, and appointments for diagnostics or follow-up may be more difficult for adults with hearing loss.

Overwhelmingly, the largest health disparity between individuals with some form of hearing loss and those who reported excellent/good hearing was in moderate and vigorous physical activity. Those with hearing loss reported greater difficulty in engaging in moderate and vigorous physical activity compared to those with excellent/good hearing. Regular physical activity has been associated with better weight control, reduction in the risk for cardiovascular disease (CVD), Type 2 diabetes, and some cancers [28]. A connection has been noted between low cardiovascular fitness and reduced hearing levels in individuals over the age of 49 in specific frequency ranges [29]. The direction of this relationship was not established though. The CDC recommends 150 min of moderate activity or 75 min of vigorous activity weekly for adults 18–65 years of age [30]. It is unlikely that this recommendation is being met by many adults with hearing loss. While this study is unable to elucidate if one of these is causal, hearing loss or the inability to exercise, it is able to recognize there is an association between the two.

All people face some barriers to engaging in regular, moderate, or vigorous activity. For example, Freene et al. (2011) [31] identified personal barriers among middle-age adults for both home-based and group-based exercise. Such barriers included low self-efficacy, low priority, and health problems that interfered with exercise. Adults with hearing loss likely face these same barriers plus more. The barriers to exercise are likely exacerbated by hearing loss. Additional barriers for individuals with hearing loss may include having to remove hearing aids to protect the electronics from sweat, concerns for safety (not being able to hear other people or traffic), inability to hear the exercise instructor or gym assistant in either quiet or noisy environments, inability to hear or understand conversation from others who are exercising nearby, embarrassment of hearing loss, or being self-conscious about wearing hearing devices. Furthermore, people with severe hearing loss may be more socially isolated and lack the social support needed to engage in regular physical activity/exercise.

Whatever the reason(s) that individuals with hearing loss report greater difficulty exercising, we know that negative health outcomes increase as inactivity increases. This may be one reason adults with hearing loss were more likely to be diagnosed with conditions such as high cholesterol, hypertension, diabetes, cancer, and stroke.

Some of these barriers to exercise could be overcome by educating health coaches and others in related fields about how to better communicate with individuals with hearing loss. Online training modules could be constructed and required for licensure through such agencies as the National Commission for Health Education Credentialing (NCHEC), National Council on Strength and Fitness (NCSF), American Council on Exercise (ACE), National Academy of Sports Medicine (NASM), International Sports Sciences Association (ISSA), and CrossFit. Targeting the importance of moderate and vigorous exercise to those with hearing loss through education and health risk messaging would be a great first step in improving the activity level for individuals with hearing loss. Furthermore, inter-professional education between physicians and audiologists is needed to better tailor and customize messages and materials regarding exercise and how to reduce barriers to exercise among those with hearing impairments.

Regarding lifestyle and prevention, some types of hearing loss can be preventable—for example, noise-induced hearing loss, loss caused by a singular or regularly occurring loud event/episode. Causes may include loud music, operation of loud machinery, or a single episode of uniquely loud stimuli (e.g., gunfire and explosions). Early and consistent hearing screenings with proper referral and follow-up may help to reduce the negative health impact of hearing loss of any kind. Improved access to, use of, and social awareness of assistive devices such as hearing aids, cochlear implants, Frequency Modulation FM systems, sound-fields, infrared systems, and induction loops may also have a positive effect on the health of individuals with hearing loss. Such primary and secondary prevention efforts would help public health experts to meet the specific hearing objectives in Healthy People 2020 [32].

### Limitations

The results of this study should be viewed with its potential limitations in mind. First, the NHIS used self-reported data. Self-reported data, especially responses gathered via personal interview, have the potential for socially desirable responses which can create bias in the results. Therefore, healthy behaviors such as exercise and preventive screenings may have been over-reported. Second, the NHIS survey used broad, general response items for participants to rate their hearing. Such loosely defined responses may have led to a lack of specificity and accuracy. This may have negatively impacted the internal validity of the results regarding classification of hearing loss. Third, the nature of the NHIS data is cross-sectional, and the statistical analyses used by the investigators of the current study preclude causal references.

Further research is needed to better understand the reasons adults with hearing loss report higher levels of specific diseases and why they have greater difficulty engaging in regular physical activity/exercise. Utilizing mixed research methods and qualitative research in particular may help to elucidate why these difficulties exist. Examining these health outcomes/behaviors in relation to variables such as age of onset of hearing loss, percent of life with hearing loss, hours of device use, support systems, possible habilitation or rehabilitation, and type of device would provide invaluable information.

Finally, more efforts are needed to improve health professionals’ awareness of and sensitivity to those with hearing loss and how hearing loss impacts health. Such efforts should include education and training for future healthcare providers, medical social workers, patient educators, health educators, and community health workers. Inter-professional workshops/seminars/trainings to improve communication with patients/clients regarding the health implications of hearing loss need to be established, and research needs to be done on how to best address this growing population.

## Figures and Tables

**Table 1:** Participant demographics: all National Health Interview Survey (NHIS) participants (2014).

Characteristics	N	%
Region of the United States		
South	12,896	(35)
West	10,073	(27)
Midwest	7809	(21)
Northeast	5919	(16)
Sex		
Female	20,299	(55)
Male	16,398	(45)
Age (M = 49, SD = 18.3)		
≤19	769	(2)
20–29	5788	(16)
30–39	6168	(17)
40–49	5861	(16)
50–59	6483	(18)
60–69	5786	(16)
≥70	5842	(16)
Marital Status		
Married—spouse in household	15,424	(42)
Never married	8602	(23)
Divorced	5184	(14)
Widowed	3480	(10)
Living with partner	2253	(6)
Separated	1044	(3)
Married—spouse not in household	624	(2)
Unknown marital status	86	(0)
Race		
White	28,526	(78)
Black/African American	5310	(15)
Asian	2168	(6)
Indian (American), Alaska Native	468	(1)
Multiple race, no primary selected	101	(0)
Level of hearing		
Excellent	17,500	(48)
Good	12,316	(34)
A little trouble	4170	(11)
Moderate trouble	1658	(5)
A lot of trouble	936	(3)

Source: Center for Disease Control and Prevention (CDC)/National Center for Health Statistics (NCHS) National Health Interview Survey, 2014, Note: N = 36,697; Percentages may not equal 100% due to rounding.

**Table 2:** Self-reported health status and disease states: all NHIS respondents (2014).

Health Status/Chronic Illness	N	(%)
**Self-Reported health status**		
Better than last year	6476	(18)
About the same as last year	26,976	(74)
Worse than last year	3191	(9)
**Body Mass Index (BMI)**		
Underweight	613	(2)
Normal	11,685	(32)
Overweight	11,963	(33)
Obese	11,905	(32)
**Self-Reported Disease States**		
Hypertension	12,396	(34)
High Cholesterol	10,928	(30)
Asthma	4769	(13)
Tinnitus	4514	(12)
Diabetes	3832	(10)
Cancer (any kind)	3448	(9)
Heart Disease	1976	(5)
Ulcer	2757	(8)
COPD	1371	(4)
Stroke (ever had)	1183	(3)
Hepatitis	1027	(3)
Long term liver condition	483	(1)

Source: CDC/NCHS, National Health Interview Survey, 2014, Note: N = 36,697; Percentages may not equal 100% due to rounding.

**Table 3:** Comparison of disease states by level of hearing loss to excellent/good hearing status.

Health Status/Disease States	A Little Trouble Hearing		Moderate/A Lot of Trouble Hearing		Deaf	
	OR	CI	OR	CI	OR	CI
Hypertension	2.530 (2.369–2.702)		3.434 (3.164–3.728)		3.377 (2.310–4.937)	
Asthma	1.273 (1.162–1.394)		1.278 (1.143–1.430)		1.298 (0.773–2.180) *	
Diabetes	2.229 (2.037–2.439)		2.731 (2.461–3.029)		3.299 (2.121–5.132)	
Cancer (any kind)	2.470 (2.251–2.710)		3.458 (3.118–3.834)		3.717 (2.375–5.819)	
Heart disease	3.191 (2.830–3.598)		5.049 (4.452–5.725)		4.960 (3.908–8.460)	
COPD	2.930 (2.554–3.362)		4.829 (4.194–5.560)		2.587 (1.199–5.582)	
Stroke	2.733 (2.356–3.171)		4.438 (3.811–5.167)		2.050 (0.833–5.044) *	
High cholesterol	2.296 (2.149–2.454)		2.884 (2.659–3.128)		1.810 (1.228–2.667)	
Ulcer	2.212 (1.994–2.452)		2.897 (2.580–3.252)		2.826 (1.682–4.750)	
Hepatitis	1.656 (1.396–1.964)		1.889 (1.548–2.304)		0.735 (0.181–0.983) *	
Tinnitus	5.431 (5.017–5.880)		8.661 (7.918–9.475)		3.897 (2.483–6.115)	
Long term liver condition	2.249 (1.787–2.831)		2.976 (2.317–3.821)		2.671 (0.843–8.461) *	
Overweight or obese	1.442 (1.323–1.529)		1.305 (1.195–1.426)		1.046 (0.702–1.557) *	
Health status compared to year ago						
better	0.896 (0.822–0.978)		0.747 (0.666–0.838)		1.329 (0.850–2.079) *	
same	0.765 (0.712–0.821)		0.700 (0.674–0.764)		0.666 (0.448–0.990)	
worse	2.137 (1.937–2.356)		2.929 (2.626–3.266)		1.597 (0.879–2.914) *	

Source: CDC/NCHS, National Health Interview Survey, 2014, Note: OR = odds ratio; CI (confidence interval) = 95%, * = Not significant at *p* = 0.05.

**Table 4:** Comparison of medical/screening behaviors by level of hearing loss to excellent/good hearing status.

Medical Behaviors/Screenings	A Little Trouble Hearing		Moderate/A Lot of Trouble Hearing		Deaf	
	OR	CI	OR	CI	OR	CI
Fasted blood sugar testing (screen for diabetes) in past 12 months	1.209 (1.129–1.294)		1.225 (1.126–1.333)		1.198 (0.809–1.774) *	
Dentist visit in last 12 months	0.802 (0.750–0.857)		0.809 (0.746–0.879)		0.756 (0.515–1.111) *	
Blood pressure checked in past 12 moths	1.377 (1.226–1.546)		1.578 (1.354–1.838)		1.561 (0.758–3.213) *	
Ever been tested for HIV—males	0.974 (0.884–1.074)		0.724 (0.641–0.817)		0.502 (0.263–0.957)	
females	0.724 (0.656–0.799)		0.491 (0.427–0.565)		0.662 (0.358–1.224) *	
Ever rec’d HPV—males	0.537 (0.340–0.847)		0.525 (0.269–1.024) *		1.470 (0.198–10.921) *	
females	0.663 (0.525–0.838)		0.502 (0.331–0.761)		0.351 (0.047–2.602) *	

Source: CDC/NCHS, National Health Interview Survey, 2014, Note: OR = odds ratio; CI (confidence interval) = 95%, * = Not significant at *p* = 0.05.

**Table 5:** Medical/screening behaviors by hearing level: All NHIS participants (2014).

Medical/Screening Behavior	Total 2014 NHIS Population (%)	Excellent/Good Hearing (%)	A Little Trouble Hearing (%)	Moderate/A Lot of Trouble Hearing (%)	Deaf (%)
**Dental Care (time since last saw a dentist)**					
Less than 12 months ago	(60)	(61)	(56)	(56)	(54)
Longer than 12 months	(38)	(38)	(43)	(42)	(42)
Never	(1)	(1)	(2)	(2)	(4)
Yes	(33)	(33)	(38)	(38)	(37)
**Hypertension (blood pressure checked by health professional in past 12 months)**					
Yes	(86)	(89)	(91)	(92)	(92)
**Flu vaccine (in past 12 months)**					
Flu shot	(42)	(40)	(54)	(60)	(61)
**HIV testing (ever been tested)**					
Males—Yes	(35)	(37)	(37)	(30)	(23)
Females—Yes	(38)	(41)	(34)	(26)	(32)
**HPV vaccine (ever received)**					
Males—Yes	(3)	(3)	(2)	(1)	(4)
Females—Yes	(11)	(11)	(8)	(6)	(4)

Source: CDC/NCHS, National Health Interview Survey, 2014.

**Table 6:** Health behaviors of all NHIS respondents: (2014).

Health Behaviors	N	(%)
**Sleep (hours per night)**		
6 h or less	11,096	(30)
7–8 h	21,439	(58)
More than 8 h	4162	(11)
**Smoking**		
Current everyday smoker	4858	(13)
Current some days smoker	1520	(4)
Former smoker	8281	(23)
Never smoker	21,838	(60)
**Consume alcohol (12+ drinks in a year)**		
Yes	23,035	(63)
No	13,356	(36)
**Frequency of drinking among regular drinkers**		
Less than 1 day per week	10,759	(37)
1–2 days	7525	(26)
3–6 days	2945	(10)
7 days	1689	(6)
**Exercise level—**		
**Moderate**		
Never engages	14,247	(39)
Engage in vigorous activity lasting at least 10 min	21,290	(58)
Unable to engage in vigorous activity	496	(1)
**Vigorous**		
Never engages	16,567	(53)
Engage in vigorous activity lasting at least 10 min	15,964	(44)
Unable to engage in vigorous activity	731	(2)

Source: CDC/NCHS, National Health Interview Survey, 2014, Note. N = 36,697; percentages may not equal 100% due to rounding.

**Table 7:** Comparison of lifestyle/health behaviors by level of hearing loss compared to excellent/good hearing status.

Health Status/Disease States	A Little Trouble Hearing		Moderate/A Lot of Trouble Hearing		Deaf	
	OR	CI	OR	CI	OR	CI
**Sleep**						
6 h or less	1.214 (1.133–1.300)		0.964 (0.883–1.053) *		0.842 (0.551–1.288) *	
7–8 h	0.731 (0.685–0.780)		0.687 (0.634–0.744)		0.620 (0.426–0.902)	
More than 8 h	1.399 (1.271–1.540)		2.232 (2.014–2.474)		3.011 (1.957–4.631)	
**Smoking**						
Current everyday smoker	1.343 (1.228–1.467)		1.083 (0.963–1.218) *		1.361 (0.819–2.260) *	
Current some days smoker	0.916 (0.775–1.082) *		0.829 (0.667–1.029) *		1.093 (0.445–2.686) *	
Former smoker	1.789 (1.666–1.922)		2.438 (2.241–2.654)		1.453 (0.949–2.226) *	
Never smoker	0.558 (0.523–0.596)		0.478 (0.440–0.518)		0.639 (0.438–0.933)	
**Consume alcohol** (12 + drinks in a year)	0.052 (0.047–0.057)		0.051 (0.046–0.058)		0.068 (0.41–0.113)	
**Frequency of drinking among regular drinkers**						
<1 day per week	0.760 (0.695–0.830)		0.792 (0.720–0.870)		0.545 (0.336–0.886)	
1–2 days per week	1.282 (1.172–1.402)		1.180 (1.071–1.300)		1.699 (1.061–2.719)	
3–6 days per week	1.091(0.963–1.236) *		1.176 (1.032–1.339)		1.188 (0.623–2.265) *	
**Exercise level**						
**Moderate (weekly—Engage in moderate activity lasting at least 10 min)**						
Never engages	1.057 (0.988–1.129) *		1.312 (1.209–1.423)		1.278 (0.872–1.871) *	
Engages	0.887 (0.830–0.947)		0.678 (0.25–0.736)		0.582 (0.398–0.851)	
Unable to engage	2.708 (2.159–3.396)		4.192 (3.322–6.290)		9.449 (4.727–18.889)	
**Vigorous (weekly—Engage in vigorous activity lasting at least 10 min)**						
Never engages	1.537 (1.437–1.643)		2.036 (1.867–2.221)		1.574 (1.063–2.330)	
Engages	0.590(0.551–0.632)		0.933 (0.359–0.431)		0.439 (0.288–0.670)	
Unable to engage	2.542 (2.103–3.074)		4.150 (3.423–5.031)		7.032 (3.642–13.578)	

Source: CDC/NCHS, National Health Interview Survey, 2014, Note: OR = odds ratio; CI (confidence interval) = 95%, * = Not significant at *p* = 0.05.

## Data Availability

Data was obtained from the Sample Adult File of the 2016 NHIS data release https://www.cdc.gov/nchs/nhis/nhis_2016_data_release.htm (accessed on 9 May 2015).
